# Vital signs following autotransfusion in liposuction and concurrent aesthetic procedures

**DOI:** 10.1590/0100-6991e-20253788-en

**Published:** 2025-08-18

**Authors:** JULIANA ALMEIDA OLIVEIRA, POLLYANA MAGALHÃES GONTIJO, JULIE STEPHANNY DE SOUZA GURGEL PARANHOS, LEANDRO COSTA GONTIJO

**Affiliations:** 1 - Universidade Federal de Minas Gerais, Programa de Pós-graduação em Cirurgia - Belo Horizonte - MG - Brasil; 2 - Instituto Mineiro de Cirurgia Plástica - Belo Horizonte - MG - Brasil; 3 - Faculdade de Ciencias Médicas de Minas Gerais, Faculdade de Enfermagem - Belo Horizonte - MG - Brasil

**Keywords:** Lipectomy, Blood Transfusion, Autologous, Cosmetic Techniques, Plastic Surgery Procedures, Vital Signs, Lipectomia, Lipoabdominoplastia, Transfusão Autóloga de Sangue, Técnicas Cosméticas, Procedimentos Cirúrgicos Plásticos, Sinais Vitais

## Abstract

**Introduction:**

Liposuction might lead to complications such as bleeding and anemia, and the volume of blood lost cannot be predicted. Autotransfusion has hemodynamic benefits and may be associated with better patient recovery.

**Objective:**

To evaluate whether Autolog IQTM impacts the vital signs of patients undergoing liposuction.

**Methods:**

A retrospective case-control study with patients undergoing liposuction from July to November 2023. Observers were blinded to data collection and analysis, and 98 patients were included and classified into an intervention group (autotransfusion during the procedure) or control group.

**Results:**

49 patients used Autolog, and 49 patients made up the control group, selected conveniently. 94 patients (96%) were women, with a mean age of 39±9.17 years and a mean weight of 26.5±3.55kg. Heart rate (HR) response in the postoperative period (MD -12, 95% CI: -19.42 to -4.58, p=0.002) and during anesthesia recovery (MD -8, 95% CI: -13.56 to -2.44, p=0.005) compared to the perioperative period favored the Autolog group. Mean arterial pressure during anesthesia recovery compared to the perioperative period (MD -25, 95% CI: -30.5 to -19.95, p<0.001); the MEWS score at hospital discharge (MD 1, 95% CI: 0.56 to 1.44, p<0.001); and HR at hospital discharge compared to postoperative (MD 10.5, 95% CI: 2.5 to 18.5, p=0.01) and anesthesia recovery (MD 8, 95% CI: 1.45 to 14.55, p=0.02) favored the control group.

**Conclusions:**

Autotransfusion showed potential benefits in immediate postoperative heart rate response and anesthesia recovery. Broader studies are needed in this population.

## RESUMO

**Introdução:**

A lipoaspiração pode ter como complicações a hemorragia e a anemia, e o volume de sangue perdido não pode ser previsto. A autotransfusão possui benefícios hemodinâmicos e pode estar associada a melhor recuperação do paciente.

**Objetivo:**

Avaliar se o Autolog IQTM impacta os sinais vitais de pacientes submetidos a lipoaspiração.

**Métodos:**

Estudo retrospectivo de caso-controle com pacientes submetidos a lipoaspiração de julho a novembro de 2023. Os observadores foram cegados para coleta e análise de dados, e 98 pacientes foram incluídas e classificadas em grupo de intervenção (autotransfusão durante o procedimento) ou controle.

**Resultados:**

49 pacientes foram usaram o Autolog e 49 pacientes compuseram o grupo controle, por seleção conveniente. 94 pacientes (96%) eram mulheres, com idade média de 39±9,17 anos e peso médio de 26,5±3,55kg. A resposta da frequência cardíaca (FC) no período pós-operatório (MD -12, IC95%: -19,42 a -4,58, p=0,002) e na recuperação da anestesia (MD -8, IC95%: -13,56 a -2,44, p=0,005) comparada ao período perioperatório favoreceu o grupo Autolog. A média da pressão arterial na recuperação anestésica comparada ao período perioperatório (MD -25, IC95%: -30,5 a -19,95, p<0,001); o escore MEWS na alta hospitalar (MD: 1, IC95%: 0,56 a 1,44, p<0,001); e a FC na alta hospitalar comparada ao pós-operatório (MD: 10,5, IC95%: 2,5 a 18,5, p=0,01) e à recuperação da anestesia (MD: 8, IC95%: 1,45 a 14,55, p=0,02) favoreceram o grupo controle.

**Conclusão:**

A autotransfusão apresentou potenciais benefícios na resposta cardíaca pós-operatória imediata e na recuperação anestésica. Necessita-se de estudos mais abrangentes nessa população.

## INTRODUCTION

Liposuction is the most popular aesthetic procedure in Brazil^1^. It aims to reshape areas of the body by removing fat deposits, especially when they persist after weight loss attempts through physical activity and dietary changes^2^ ^,^ ^3^. It can be performed on a wide range of regions, such as thighs, neck, waist, back, chin, legs, and ankles. In addition, it can be conducted in conjunction with other procedures, such as mammoplasty or abdominoplasty^2^.

Liposuction brings several benefits, such as reduction of body weight^4^ ^,^ ^5^, fat, waist circumference, leptin levels4, total serum cholesterol levels, and reduction of low-density lipoprotein (LDL)4 and elevation of high-density lipoprotein (HDL)^6^. Thus, an increase is expected over the years^7^, which can already be observed in the reports of the International Society of Aesthetic Plastic Surgery (ISAPS), with a gradual increase in the number of surgeries performed annually^1^. In addition, studies have associated liposuction with a reduction in systolic blood pressure^8^ ^,^ ^9^ and greater self-confidence and motivation to lose weight^10^, as well as with mental health benefits^4^. Improving quality of life and speeding up patient recovery to enable a quicker return to normal activities is essential.

Most complications result from lack of adherence to safety guidelines^11^, with excessive use of local anesthesia in large-volume liposuction, inadequate patient selection, insufficient postoperative surveillance, and aspirated volume above 7% of body weight^12^. Once these issues are controlled, other factors such as anemia and hemorrhage should be considered^12^. Thus, autologous transfusion systems, which have been used for more than 40 years^13^, can be an effective solution to reduce costs and improve patient recovery.

Autotransfusion reduces the need for allogeneic transfusions, offering a low risk of inducing pathological responses^13^. During surgery, especially in procedures with unpredictable blood loss, autotransfusion is a relevant method to maintain safety^14^. The need for transfusion increases with the amount of tissue removed, such as in major aspirations in obese patients, and autologous blood donation is widely accepted by patients^15^.

Post-liposuction recovery can be time-consuming, and when the patient does not stay in the hospital overnight, follow-up at home is necessary due to pain and residual effects of anesthesia and/or analgesia^16^. In addition, blood loss can be significant, especially between the 3^rd^ and 7^th^ postoperative days, emphasizing the importance of the patient’s clinical status at the time of hospital discharge^17^. The Modified Early Warning Score (MEWS), used to monitor complications, is an important tool for identifying deteriorations in patients’ health through the monitoring of vital signs^18^. 

This retrospective case-control study aims to evaluate vital signs in patients undergoing liposuction and concomitant procedures, comparing those who used the Medtronic autotransfusion and lavage system with those who did not.

## METHODS

### Objective and study description

We conducted a retrospective study to evaluate the impact on vital signs of the use of AutoLog IQTM associated with the Medtronic lavage set^19^, during liposuction and other associated procedures. We included all patients who underwent liposuction at the Instituto Mineiro de Cirurgia Plástica (IMCP) in Belo Horizonte, Minas Gerais State, Brazil.

The use of AutoLog IQTM in IMCP was initiated in July 2023, with data collection from patients operated from July to November 2023, by the same surgical team. All patients approached were eligible and included in the study. Randomization was not possible due to the different needs of patients in cosmetic surgeries and the cost of AutoLog IQTM. The Ethics in Research Committee (CAAE: 72973923.0.0000.5125) issued it approval on December 11, 2023, following the guidelines of the Declaration of Helsinki^20^. 

The intervention group consisted of patients who accepted the use of AutoLog IQTM. The device was used during the procedure, involving the removal and collection of fat, followed by washing with saline solution and processing of the washed liquid. The control group consisted of patients who did not use the system.

### Eligibility criteria

We included patients who met all the following eligibility criteria: (1) patients who were clinically stable before the procedure^21^; (2) undergoing liposuction; and (3) with any outcome of interest recorded. The use of the autotransfusion system depended on the financial conditions and consent of the patients. The choice of the technique did not interfere with the safety and quality of the care provided. All patients were previously evaluated by a plastic surgeon for clinical and physical evaluation. We excluded patients under 18 years of age, with hematological disorders, and with surgical risk greater than II, according to the classification of the American Society of Anesthesiology (ASA)^22^. 

The outcomes of interest were (1) heart rate (HR), (2) capillary blood oxygen saturation, (3) mean arterial pressure (MAP), and (4) MEWS at four different time points, namely, preoperative (baseline), immediately postoperatively, after recovery from anesthesia, and at hospital discharge. We compared the last three scenarios with each other and with baseline data to assess the patients’ vital signs response.

The MEWS is a tool designed to prevent late interventions or transfers of critically ill patients, and is useful in inpatient surgical patients. It assesses systolic blood pressure, heart rate, temperature, respiratory rate, urine output, and level of consciousness. Scores usually range from 0 to 3, and the higher the score, the greater the patient’s risk.

### Data extraction and subgroup analysis

The authors (JAO and JSSGP) blindly collected and analyzed the data, with data verification by the two team members. As a subgroup, we compared the information for patients undergoing liposuction alone with those undergoing more procedures in the same surgery. No other subgroup analysis was possible.

### Statistical analysis

Statistical analysis was conducted using RStudio (PBC, Boston, MA) to determine the statistical value of the differences observed between the intervention and control groups. As measures of effect for binary outcomes, we used odds ratios (OR). For continuous outcomes, we used the mean and standard deviation. The results were considered statistically significant when p<0.05. We calculated 95% confidence intervals to provide an estimate of the accuracy of the observed effects. The random effects model of DerSimonian and Laird^23^ was used for all outcomes to reduce sampling bias. No patients were excluded. We applied the Strengthening the Reporting of Observational Studies in Epidemiology (STROBE)^24^ checklist to ensure the quality of the case-control study. 

## RESULTS

After applying the eligibility criteria, we included 98 patients in the study (Figure 1). Of these, 49 patients (50%) used Autolog IQTM, of which three were men (6%). In the intervention group, five (10.2%) patients underwent liposuction only. The other 44 had other procedures performed concomitantly. Of the 15 patients (30.6%) who underwent mastopexy, one (2%) had associated torsoplasty, three (6%) had associated argoplasma, and one (2%) had associated sapphire. In the control group, only one (2%) male was included. Of the 49 patients, five (10.2%) underwent liposuction alone, two (4%) had it associated with argoplasma, and one (2%) with the UGRAFT technique ^28^. Patients’ characteristics can be seen in more detail in Table 1.

**Table 1:** Characteristics of the patients included and the procedure by group.

		TOTAL (n= 98)	AUTOLOG IQ (n= 49)	CONTROL (n= 49)
Age (years)^a^	Total	39 ± 9.17	40.2 ± 9.02 (range: 23 to 64)	39.3 ± 9.39 (range: 25 to 65)
Weight (kg)ª	Total	70.65 ± 10.38	73.7 ± 11.23 (range: 52 to 99)	69.6 ± 9.1 (range: 56 to 102)
BMI (kg/m²)ª	Total	26.5 ± 3.55	27.08 ± 3.9 (range: 21.5 to 37.3)	25.8 ± 3.05 (range: 20.9 to 32.6)
	Normal	23.2 ± 1.19	23.3 ± 1.21 (range: 21.5 to 24.9)	23.2 ± 1.19 (range: 20.9 to 25)
	Overweight	27.5 ± 1.07	27.6 ± 1.04 (range: 25.6 to 29.4)	27.3 ± 1.16 (range: 25.1 to 29.7)
	Obese	32.8 ± 2.05	33.4 ± 2.11 (range: 30.9 to 37.3)	31.5 ± 0.98 (range: 30.5 to 32.6)
Skin color No. (%)	White	64 (65.3%)	36 (73.6%)	28 (57.1%)
	Brown	28 (28.6%)	13 (26.5%)	15 (30.6%)
	Black	6 (0.6%)	0 (0%)	6 (12.2%)
Associated procedures No. (%)	Abdominoplasty	41 (41.8%)	20 (40.8%)	21 (42.9%)
	Bodytite	26 (26.5%)	17 (34.7%)	9 (18.4%)
	Mastopexy	38 (38.7%)	15 (30.6%)	23 (46.9%)
	Morpheus	18 (18.4%1)	15 (30.6%)	3 (6.1%)

For all evaluations, the total value and/or percentage was provided, except: ª Mean value and standard deviation. * The analysis is presented for the groups (patients who used the autotransfusion system versus those who did not) and their respective subgroups. BMI: Body Mass Index.

Most patients were overweight or obese. During the study, there were no deaths or serious outcomes, nor any adverse effects associated with treatment.

### O2 saturation, heart rate, mean arterial pressure, and MEWS score

Vital signs were assessed at three different time points: immediately after the postoperative period, after recovery from anesthesia, and after hospital discharge. The MEWS score at hospital discharge significantly demonstrated a lower risk of deterioration in the control group than in the intervention one (MD 1, 95% CI 0.56 to 1.44, p<0.001). The MEWS score was not significant in the immediate postoperative period (MD 0, 95% CI -0.75 to 0.75, p>0.99) and after recovery from anesthesia (MD 0, 95% CI -0.43 to 0.43, p>0.99). O2 saturation, heart rate, and MAP showed non-significant results, which can be better visualized in Table 2.

**Table 2:** Vital signs of patients at three different moments.

	Total	Autolog IQ (n= 49)	Control (n= 49)	Statistical analysis*		
				MD	95% CI	p-value
Immediate postoperative period						
O2 Saturation	98 ± 2.71 (n= 84)	98 ± 2.6 (n= 36)	98 ± 2.81 (n= 48)	0	-1.16 to 1.16	>.99
Cardiac frequency	78 ± 13.75 (n= 84)	79 ± 10.29 (n= 36)	79 ± 14.99 (n= 48)	0	-5.41 to 5.41	>.99
MAP	77 ± 12.37 (n= 84)	76 ± 10.92 (n= 36)	77 ± 12.85 (n= 48)	-1	-6.09 to 4.09	0.7
MEWS	2.5 ± 1.7 (n= 62)	2 ± 1.26 (n= 26)	2 ± 1.18 (n= 36)	0	-0.75 to 0.75	>.99
After anesthetic recovery						
O2 Saturation	98 ± 1.14 (n= 97)	98 ± 0.63 (n= 48)	98 ± 1.39	0	-0.43 to 0.43	>.99
Cardiac frequency	85 ± 11.74 (n= 97)	83.5±11.59 (=48)	87 ± 12	-3.5	-8.19 to 1.19	0.14
MAP	79 ± 9.38 (n= 97)	78 ± 7.95 (n= 48)	79 ± 10.65	-1	-4.73 to 2.73	0.6
MEWS	1 ± 1.38 (n= 96)	1 ± 1.12 (n= 48)	1 ± 1.05 (n= 48)	0	-0.43 to 0.43	>.99
At hospital discharge						
O2 Saturation	97 ± 1.86 (n= 94)	97.5 ± 1.91 (n=48)	97 ± 1.82 (n= 46)	0.5	-0.25 to 1.25	0.19
Cardiac frequency	94.5 ± 13.5 (n= 94)	96 ± 13.31 (n= 48)	94 ± 13.68 (n= 46)	2	-2.80 to 6.80	0.41
MAP	81 ± 9.61 (n= 94)	82 ± 10.77 (n= 48)	80 ± 8.26 (n= 46)	2	-1.87 to 5.87	0.31
Escore de MEWS	1 ± 1.08 (n= 95)	2 ± 1.09 (n= 48)	1 ± 1.08 (n= 47)	1	0.56 to 1.44	<0.001

For all evaluations, the total value and/or percentage was provided, except: ª Mean value and standard deviation. * The analysis is presented for the groups (patients who used the autotransfusion system versus those who did not) and their respective subgroups. MAP: Mean arterial pressure.

### Immediate postoperative period compared to baseline (preoperative)

Heart rate response significantly favored the Autolog group (MD -12, 95% CI -19.42 to -4.58, p=0.002). We found non-significant data for O2 saturation (MD -0.05, 95% CI -1.76 to 0.76, p=0.44) and MAP (MD -5, 95% CI -11.36 to 1.36, p=0.12). More details of the statistical analysis can be seen in Table 3.

**Table 3:** Patients’ vital signs in the immediate postoperative period (difference from the immediate preoperative period).

	Total	Autolog IQ (n= 49)	Control (n= 49)	Statistical analysis*		
				MD	95% CI	p-value
O2 saturation	0±2.94	-0.5±2.69 (range: -7 to 5; n=38)	0±3.06 (range: -9 to 3)	-0.5	-1.76 to 0.76	0.44
Cardiac frequency	1±17.15	-7±13.9 (range: -29 to 26; n=38)	10±17.39 (range: -27 to 44)	-12	-19.42 to 4.58	0.002
MAP	-13±14.71	-15±15.80 (range: -44 to 16; n=38)	-12±13.94 (range: -48 to 20)	-5	-11.36 to 1.36	0.12

For all evaluations, the total value and/or percentage was provided, except: ª Mean value and standard deviation. * The analysis is presented for the groups (patients who used the autotransfusion system versus those who did not) and their respective subgroups. MAP: Mean arterial pressure.

### Anesthesia recovery period compared to preoperative and immediate postoperative

We had two significant findings: heart rate favoring the Autolog group (MD -8, 95% CI -13.56 to -2.44, p=0.005) and mean arterial pressure favoring the Control group (MD -25, 95% CI -30.5 to -19.95, p<0.001), both when comparing the difference after recovery from anesthesia with the perioperative period. O2 saturation was not statistically significant (MD 0, 95% CI -0.65 to 0.66, p>0.99). When recovery from anesthesia was compared to the postoperative period, O2 saturation (MD 0, 95% CI -1.13 to 1.13, p>0.99), heart rate (MD 5, 95% CI -3.51 to 13.51, p=0.25), and mean arterial pressure (MD 0, 95% CI -4.88 to 4.66, p>0.99) did not show statistical significance. More details can be found in Table 4.

**Table 4:** Vital signs immediately after recovery from anesthesia (difference from the immediate preoperative and postoperative periods).

	Total	Autolog IQ (n= 49)	Control (n= 49)	Statistical analysis*		
				MD	95% CI	p-value
Post-anesthesia recovery vs. immediate preoperative phase						
O2 saturation	0 ± 1.76	0 ± 1.54 (range: -3 to 3; n= 49)	-0.5 ± 1.89 (range: -6 to 2; n= 36)	0	-0.65 to 0.65	>.99
Cardiac frequency	5 ± 13.50	0 ± 12.79 (range: -19 to 40; n= 49)	8.5 ± 13.74 (range: -27 to 31)	-8	-13.56 to -2.44	0.005
MAP	-13 ± 12.94	(range: -41 to 12; n= 35)	-10 ± 13.97 (range: -45 to 23)	-25	-30.05 to -19.95	<0.001
Post-anesthesia recovery vs. immediate postoperative phase						
O2 saturation	0 ± 2.39	0 ± 2.23 (range: -2 to 5; n= 35)	0 ± 2.52 (range: -2 to 7; n= 48)	0	-1.13 to 1.13	>.99
Cardiac frequency	-1 ± 13.88	4 ± 22.98 (range: -16 to 50; n= 37)	-2 ± 12.85 (range: -37 to 31; n= 48)	5	-3.51 to 13.51	0.25
MAP	0 ± 11.48	-1 ± 12.53 (range: -23 to 23; n= 35)	1 ± 10.38 (range: -45 to 23; n= 48)	0	-4.88 to 4.66	>.99

For all evaluations, the total value and/or percentage was provided, except: ª Mean value and standard deviation. * The analysis is presented for the groups (patients who used the autotransfusion system versus those who did not) and their respective subgroups. MAP: Mean arterial pressure.

### Hospital discharge compared to baseline, immediate postoperative period, and anesthesia recovery period

We found no statistical relevance in vital signs at hospital discharge when compared with the preoperative period. The control group was statistically favored when the heart rate at hospital discharge was compared with the postoperative period (MD 10.5, 95% CI 2.5 to 18.5, p=0.01) and the anesthesia recovery period (MD 8, 95% CI 1.45 to 14.55, p=0.02). O2 saturation and mean arterial pressure did not show statistically significant findings when hospital discharge was compared with the postoperative and recovery periods from anesthesia. Other findings are available in the Supplementary Material.

### Subgroup analysis

In patients who underwent liposuction alone, O2 saturation, heart rate, MAP, and MEWS score could not be evaluated immediately after the postoperative period, due to the low amount of data in the Autolog group (n=1). There was no difference in recovery from anesthesia (five patients in each group) for the outcomes of O2 saturation (MD 0, 95% CI -1.05 to 1.05, p>0.99), HR (MD -1, 95% CI -18.75 to 16.75, p=0.91), MAP (MD 0.8, 95% CI -16.15 to 17.75, p=0.93), and MEWS score (MD 0, 95% CI -1.49 to 1.49, p>0.99). There was also no difference in the assessment of vital signs at hospital discharge (n=5 for each group) regarding O2 saturation (MD -0.1, 95% CI -2.31 to 2.11, p=0.93), heart rate (MD 1.2, 95% CI -14.2 to 16.6, p=0.88), MAP (MD -3.2, 95% CI -13.09 to 6.69, p=0.53), and MEWS score (MD 0.2, 95% CI -1.1 to 1.5, p=0.76).

## DISCUSSION

Autolog IQTM is indicated during surgical procedures with significant blood loss^19^ ^,^ ^21^, for the processing and reinfusion of autologous blood, ensuring a high quality of the recovered blood, with hematocrit of the washed product between 59-65%, and removal of 98% of heparin and 99% of fat^19^. The removal of these blood components increases safety, by reducing the risk of toxicities, coagulopathy, among others^21^. The device offers efficient recovery rates, with standard wash cycles of approximately 3.4 minutes, quick wash of 2.25 minutes, and emergency of 1.45 minutes, processing a volume of 135mL per cycle^19^. It is noteworthy that most studies are carried out in cardiac, vascular, or orthopedic surgery^21^. 

Our findings demonstrated that the group using Autolog had a better heart rate response in the postoperative period and in the recovery from anesthesia compared with the perioperative period. While the control group had better mean arterial pressure at recovery from anesthesia compared with the perioperative period, MEWS score at discharge and heart rate at hospital discharge compared with the postoperative period and recovery from anesthesia.

It was not possible to associate characteristics of the procedure, such as aspirated volume, with the outcomes. Some studies have found no association between aspirated volume and calculated blood loss, even in large-volume liposuction^29^ ^-^ ^39^, especially if the tumescent method is applied^39^ ^-^ ^47^, the method chosen in our study. In addition, it is expected that in overweight patients, the aspirated volume will be higher, and the blood-to-fat ratio will be lower, without affecting the safety of the surgery^48^. Therefore, in this study, we did not seek to correlate the data with the aspirated volume or to assess such difference between groups. In addition, the absence of randomization, which can result in heterogeneity in groups’ characteristics, increases a confounding factor (socioeconomic status), since the patients needed to have financial conditions to afford not only the procedure but also Autolog. 

Tachycardia is related to a higher postoperative risk of death and adverse events^49^, as well as heart rate variability^50^. Both variables displayed better results in the intervention group in the postoperative period and in the recovery from anesthesia. However, the control group showed better rates at hospital discharge compared with the postoperative period and the anesthesia recovery period. Another study showed that complications were related to greater heart rate variability and a tendency for the ratio of low frequency to high frequency on the second postoperative day^50^ in patients undergoing abdominal surgery. Therefore, hospital discharge data should be better evaluated with future studies. 

We could not correlate findings such as hypotension, another indicator of mortality, with the outcomes, due data scarcity. However, the control group showed a better response in mean arterial pressure in recovery from anesthesia.

The control group also had a better MEWS score at hospital discharge. This finding should be interpreted with caution, since studies have observed that the combination of MEWS with the post-anesthesia visit did not reduce mortality, and that MEWS should not be interpreted in isolation^51^. The score is a way to improve surgical safety, preventing delays in intervention or transfer of critically ill patients^18^. Therefore, since both groups had low scores, this finding may not be clinically significant. Another study^52^ on hospital cardiorespiratory arrest in surgical patients showed that MEWS is related to higher chances of death when increased at admission, 24 hours before the event, on the day of the event, and with a maximum score on the day of the event. 

The benefits of autologous transfusion of blood extracted from the fat removed during surgery are: (1) no need to remove 1 to 3 units of blood from the patient two weeks before the procedure; (2) no risks of adverse effects related to blood transfusion; and (3) reduction of hospital expenses. Several national and international studies have shown significant cost savings with the use of autologous transfusion compared with allogeneic blood products. Although the unit cost has already been considered high (US$ 338 versus US$ 210), there is a lower risk of adverse reactions^53^. More recent studies show a 45% saving in total cost, reducing expenses from US$ 114,523 to US$ 63,252^54^ ^,^ ^55^. In addition, in cardiac and orthopedic surgeries, there is an additional saving of US$ 55 to US$ 112 due to the increase in the costs of blood products^56^ ^-^ ^58^. These data reinforce that despite the higher initial investment in some contexts, autotransfusion can be advantageous by reducing transfusion complications and the use of hospital resources^55^.

Moreover, autologous transfusion provides a safer environment for a procedure related to blood loss, which cannot be predicted. We emphasize that a retrospective study by our research group with some of the patients in this study found no difference in the rates of complications and laboratory tests in patients who underwent liposuction^59^. However, the sample consisted of only 36 patients^59^.

## CONCLUSION

This study identified potential benefits of the use of autotransfusion systems in liposuction in relation to heart rate response in the immediate postoperative period and recovery from anesthesia. However, the impact on blood pressure and other vital signs requires further investigation, and the use of the system should be evaluated according to patients’ individual profiles. Further studies, with larger sample sizes and randomization, are needed to validate these findings.
